# PP55 First Income-Based Study Of Quality Of Life And Wellbeing In A Brazilian Non-Governmental Organization Using The EQ-Health and Wellbeing Short Version (EQ-HWB-S) Instrument

**DOI:** 10.1017/S0266462325102742

**Published:** 2025-12-29

**Authors:** Jose Evangelista, Andressa Braga, Ana Santos, Marisa Santos

## Abstract

**Introduction:**

Quality of life is crucial for assessing the impact of health and social care interventions, particularly in economically vulnerable contexts. The Dara Institute, a Brazilian non-governmental organization, uses an integrated approach to combat poverty. This study applied the EQ-HWB-S instrument to evaluate the quality of life of families served by the Institute by analyzing its relationship with income before and after intervention.

**Methods:**

Using a hybrid study design, including cross-sectional and cohort analyses, the EQ-HWB-S instrument was applied to 100 families served by the Dara Institute in Brazil before and after the intervention, enabling an analysis of wellbeing and quality of life data based on income. Microsoft Excel and Python were used for data tabulation and statistical analysis, respectively.

**Results:**

A total of 100 individuals, each representing their family served by the Institute, were included. According to the Brazil Criteria (socioeconomic status measurement score), 41 were classified as category C and 58 as D/E. In EQ-5D-3L, category C scored 0.787 (interquartile range [IQR] 0.692–0.801) and D/E scored 0.787 (IQR 0.695–0.801). In EQ-5D-5L, category C scored 0.762 (IQR 0.687–0.817) and D/E scored 0.754 (IQR 0.733–0.817). In EQ-HWB-S, category C scored 0.605 (IQR 0.457–0.865) and D/E scored 0.594 (IQR 0.449–0.743).

**Conclusions:**

Although income differences influenced EQ-5D-3L, EQ-5D-5L, and EQ-HWB scores, interquartile ranges suggested a moderate impact on quality of life outcomes measured by EQ-HWB-S. Lower-income individuals (D/E) had slightly lower EQ-HWB-S scores than those in category C, reflecting additional challenges despite intervention. The EQ-HWB-S demonstrated potential for evaluating quality of life and wellbeing in at-risk populations.

